# Adult tonsillectomy: postoperative pain depends on indications^☆^

**DOI:** 10.1016/j.bjorl.2015.11.010

**Published:** 2016-02-16

**Authors:** Olaf Zagólski, Mariusz Gajda, Paweł Stręk, Michael John Kozlowski, Artur Gądek, Jerzy Nyzio

**Affiliations:** a St. John Grande's Hospital, ENT Day Surgery Department, Kraków, Poland; b Jagiellonian University Medical College, Department of Histology, Kraków, Poland; c Jagiellonian University Medical College, Department of Otolaryngology, Kraków, Poland; d University Hospital, Department of Orthopedics and Rehabilitation, Kraków, Poland

**Keywords:** Palatine tonsils, Tonsillectomy, Laser tonsillectomy, Tonsillitis, Pain, Nerve fibers, Tonsilas palatinas, Tonsilectomia, Tonsilectomia a laser, Tonsilite, Dor, Fibras nervosas

## Abstract

**Introduction:**

Intense pain is one of the most important postoperative complaints after tonsillectomy. It is often described by patients as comparable to the pain that accompanies an acute tonsillitis. Although recurrent tonsillitis is the most frequent indication for surgery, many tonsillectomies are performed due to other indications and these patients may be unfamiliar with such pain.

**Objective:**

To verify whether individuals with recurrent tonsillitis experience different post-tonsillectomy pain intensity than those with other indications for surgery, with no history of episodes of acute tonsillitis.

**Methods:**

A total of 61 tonsillectomies were performed under general anesthesia, using a potassium titanyl phosphate (KTP) laser (to eliminate the potential influence on the study results of forceful dissection of fibrotic tonsils in patients with history of recurrent tonsillitis) and multiple ligations of blood vessels within the tonsillar beds. The patients received 37.5 mg Tramadoli hydrochloridum + 325 mg Paracetamol tablets for 10 days. Postoperative variables included the duration of hospital stay, postoperative hemorrhage and readmission rate. The patients reported pain intensity on consecutive days, pain duration, weight loss on postoperative day 10, character, intensity and duration of swallowing difficulties, and the need for additional doses of painkillers. Healing was also assessed. Capsular nerve fibers were histologically examined in the resected tonsils by immunostainings for general and sensory markers.

**Results:**

Indications for the surgery were: recurrent acute tonsillitis (34 patients), no history of recurrent tonsillitis: focus tonsil (20) and intense malodour (7). Pain intensity on postoperative days 3–4 and incidence of readmissions due to dehydration were significantly higher in the group with no history of recurrent tonsillitis. No significant differences in relative densities of protein gene product (PGP) 9.5- and calcitonin gene-related peptide (CGRP)-immunoreactive nerve fibers were observed.

**Conclusion:**

Patients with recurrent tonsillitis qualified for tonsillectomy reported lower pain intensity than those without recurrent tonsillitis and the pain scores were unrelated to nerve fibers density.

## Resumo

**Introdução:**

Dor intensa é uma das queixas mais importantes no pós-operatório de uma tonsilectomia. Com frequência, essa dor é descrita pelos pacientes, como comparável à dor que acompanha a tonsilite aguda. Apesar da tonsilite recorrente ser a indicação mais frequente para cirurgia, muitas tonsilectomias são realizadas por outras indicações, e esses pacientes podem não estar familiarizados com essa dor.

**Objetivo:**

Verificar se indivíduos com tonsilite recorrente apresentam diferenças na intensidade dolorosa pós-tonsilectomia *vs.* pacientes com outras indicações para cirurgia, sem histórico de episódios de tonsilite aguda.

**Método:**

Foram realizadas 61 tonsilectomias sob anestesia geral, com o uso de um laser potassium titanyl phosphate (KTP) (para que fosse eliminada uma possível influência de uma dissecção agressiva das tonsilas fibrosadas em pacientes com história de tonsilite recorrente), e hemostasia através de ligaduras de vasos sanguíneos nos leitos tonsilares. Os pacientes foram medicados com 37,5 mg de cloridrato de tramadol + 325 mg de paracetamol (comprimidos) durante 10 dias. As variáveis pós-operatórias foram tempo de internação hospitalar, hemorragia e percentual de readmissão. Os pacientes forneceram informações sobre a intensidade da dor em dias consecutivos, duração da dor, perda de peso corpóreo no dia 10 do pós-operatório, intensidade e duração da dificuldade de deglutição, e necessidade de doses adicionais de analgésicos. A velocidade de cicatrização também foi avaliada. Fibras nervosas capsulares foram examinadas histologicamente nas tonsilas resecadas com o uso de imunocorantes para marcadores de fibras nervosas gerais e de sensibilidade.

**Resultados:**

As indicações para a cirurgia foram: tonsilite aguda recorrente (34 pacientes), ausência de história de tonsilite recorrente – Tonsilite focal (20) e halitose (7). A intensidade da dor nos dias 3-4 do pós-operatório e a incidência de reinternações em decorrência de desidratação foram significativamente mais altas no grupo sem história de tonsilite recorrente. Não foram observadas diferenças significantes nas densidades relativas de fibras nervosas imunorreativas para protein gene product (PGP) 9.5 e calcitonin gene-related peptide (CGRP).

**Conclusão:**

Os pacientes com tonsilite recorrente e qualificados para tonsilectomia informaram menor intensidade da dor em relação aos pacientes sem histórico se tonsilite recorrente, e os escores para dor não apresentaram relação com a densidade das fibras nervosas.

## Introduction

Intense pain is one of the most important postoperative complaints after tonsillectomy, and in 20% of patients it becomes the reason for a hospital revisit, often due to subsequent dehydration.^1^, ^2^, ^3^ Pain intensity differs depending on the surgical technique^2^, ^4^, ^5^ and the type of pharmacological pain treatment.^6^, ^7^ There are different patterns of postoperative pain after tonsillectomy; most frequently, pain presents a decreasing character, but some patients report increasing pain scores during the first few postoperative days.^8^ In some individuals, pain intensity decreases a week after the surgery and in others it persists for more than two weeks.^8^ There is also a group of patients suffering from a very high level of pain from the beginning, which decreases only gradually.^8^ Nevertheless, the majority of adult patients undergoing tonsillectomy can be appropriately advised pre-operatively regarding the probable duration of postoperative pain and the time when they can expect to resume work and normal swallowing.^9^

Postoperative pain after tonsillectomy is frequently described by patients as comparable to the pain that accompanies an episode of acute tonsillitis. Most of the patients qualified for tonsillectomy had experienced such pain numerous times before the surgery, since chronic tonsillitis, defined by American Academy of Otolaryngology – Head and Neck Surgery criteria, as chronic tonsillithiasis or recurrent acute tonsillitis (disabling sore throat episodes five or more times per year, and symptoms for at least a year),^10^ constitutes the chief indication for adult tonsillectomy.^11^ Other indications are: tonsillar hypertrophy and suspected neoplasm.^12^, ^13^ However, a number of tonsillectomies are performed due to the following indications, often established by otorhinolaryngologists together with non-ENT physicians: focus-tonsil,^14^ resulting in rheumatoid arthritis, psoriasis vulgaris, pustulosis palmaris and plantaris, and/or sternocostoclavicular hyperostosis,^14^, ^15^ as well as tonsillitis-induced immunoglobulin A nephropathy.^16^ In selected cases, intense malodour can also be the indication for tonsil removal.^17^ These patients usually do not report a history of recurrent tonsillitis and may be unfamiliar with intense pharyngeal pain before they are qualified for tonsillectomy.^14^, ^15^, ^18^

Inflammatory processes have been found to influence sensory innervation in various organs and tissues.^19^, ^20^, ^21^, ^22^, ^23^ Experimental studies have shown that inflammation is generally accompanied by abnormal sprouting of peripheral sensory and autonomic nerve fibers and it significantly intensifies pain sensation.^20^, ^21^, ^22^ On the contrary, inflammation can also be associated with atrophy, apoptosis and necrosis of the sensory nerve fibers, leading to neuropeptide-mediated neuropathy in salivary glands.^19^

In this study we set out to verify: (1) whether individuals with recurrent tonsillitis in anamnesis experience different post-tonsillectomy pain intensity than those with other indications for surgery and no history of recurrent tonsillitis, and (2) to determine whether densities of capsular nerve fibers in resected tonsils differ between these groups.

## Methods

Prior to examination, all the participants signed written informed consent for their participation in the study. The research plan was approved by the institutional research committee and the local medical ethics committee (75/KBL/OIL/2010). The tenets of the Helsinki declaration were followed.

The study sample size was estimated with the use of a minimum expected difference of pain scores of 2, an estimated standard deviation of the variable of 1.5 with a resulting standardized difference of 1.3, and a desired test power of 0.8. A total of 61 tonsillectomies were performed during the period of January 2013–March 2014. There were 33 female and 28 male patients, aged 20–40 years (mean = 29.0; SD = 6.6). This study did not analyze patients in whom tonsillectomy was performed due to upper airway obstruction secondary to tonsillar hypertrophy, as all these procedures were combined with laser-assisted uvulopalatoplasty. General anesthesia induction was achieved with 1.0–2.0 mg/kg fentanyl and 1.0–2.0 mg/kg propofol 0.6–1.0 mg/kg. Rocuronium was used as a muscle relaxant for endotracheal intubation. Anesthesia was maintained by sevoflurane in an O_2_/air 50% mixture and intermittent positive pressure ventilation. Intravenous 5.0–10.0 mg morphine and 50.0–100.0 mg ketoprofen were used for analgesia.

Surgery was performed with potassium titanyl phosphate laser (KTP/532 AMS Aura XP – San Jose, CA, United States). The laser power was set at 15 W. Multiple ligations (Vicryl 4-0; Ethicon, Johnson & Johnson – New Brunswick, NJ, United States) of blood vessels within tonsillar beds were applied. Difficult to access parts of the pillars, adjacent to the tongue base, were cauterized with the laser in order to prevent postoperative bleeding. Excised tonsils were submitted for histological examination.

A single dose of intravenous steroids was administered at the time of surgery. Each patient received a single intravenous dose of 150.0 μg/kg (maximum dose 8.0 mg) dexamethasone sodium phosphate at the time of surgery, as well as three subsequent daily oral doses of 37.5 mg tramadol + 325.0 mg paracetamol, and in addition four daily 500.0 mg tablets of paracetamol for ten days. Further, up to one tablet of 50.0 mg tramadol daily was allowed. No allergy to the drugs was reported. All operations were carried out by a single surgeon. After surgery, the patients had at minimum an overnight hospital recovery.

All the patients operated on during the study period were invited to participate in the prospective, observational, questionnaire based study, and they accepted the invitation. They were followed-up for three weeks.^24^ Data collected included patient variables such as age, gender, medical history, and the indication for surgery. Postoperative variables studied included duration of hospital stay, postoperative hemorrhage and readmission rate, as well as other complications occurring during recovery from surgery. Re-admission was necessary when a patient could not drink enough fluids and required intravenous rehydration. The participants kept a daily log to assess postoperative symptoms and reported them by completing a questionnaire during follow-up visits on postoperative days four, ten, and 21. They answered a set of questions concerning pain intensity on consecutive days, pain duration, body weight loss on an empty stomach noted before tonsillectomy and on postoperative day ten (after the period of the most aggravated swallowing disorders), character and intensity of swallowing difficulties and their duration, as well as additional doses of analgesics. Postoperative maximum pain on swallowing was rated on a subjective scale of 1–5, with 1 indicating ‘no pain’ and 5 ‘severe pain’.^25^ Swallowing difficulties were rated on a scale 1–4: 1 – mild swallowing disorders, drinking unaffected; 2 – moderate difficulties eating and drinking; 3 – marked difficulties eating and drinking; 4 – serious difficulties eating and drinking.

On postoperative days four and ten, the pharynx of each patient was examined to assess the healing process, rated on a five-point scale: 4 – redness and edema of vast regions of pharyngeal mucosa, including the uvula; 3 – edema of the uvula with or without redness and/or edema of anterior pillar mucosa; 2 – redness and edema of peritonsillar tissues excluding the uvula; 1 – redness of anterior pillar mucosa without edema; 0 – normal mucosa, without redness or edema.^26^

The participants were informed about the scientific significance of the reliability of the data they were supplying. None of the patients missed the follow-up.

In order to verify whether the differences in pain intensity between the groups could result from changes of the tonsil innervation caused by repeated acute and subsequent chronic inflammation, histological examination of the densities of nerve fibers in the dissected tonsils was performed.

Both dissected tonsils were fixed overnight in 10% buffered formalin and then rinsed in phosphate buffered saline (PBS) and immersed in 25% sucrose solution. Regions of the tonsils containing the capsule were further processed. Tissue blocks were mounted in optimal cutting temperature (OCT) compound and snap-frozen. Ten-micrometer-thick cryosections were cut and thaw-mounted on poly-l-lysine-coated slides.

The sections were subjected to indirect immunofluorescence staining.^27^ Briefly, a pre-incubation step with 10% normal goat serum was applied for 40 min. The sections were incubated overnight with primary rabbit antibodies raised for general nerve fiber marker – protein gene product 9.5 (PGP 9.5; AB1761, Chemicon – Temecula, CA, United States; 1:2000) and sensory marker – calcitonin gene-related peptide (CGRP; AB5920, Chemicon; 1:4000). Subsequently, secondary incubations were applied using Cy3-conjugated goat anti-rabbit serum (111-165-144, Jackson IR – West Grove, PA, United States; 1:500) for 2 h. The sections were mounted with Vectashield medium (H-1000 – Vector, Burlingame, CA, United States).

The sections were examined using an Olympus BX-50 (Olympus – Tokyo, Japan) epifluorescence microscope equipped with appropriate filter set U-MNG for Cy3 visualization. Digital images were acquired by Olympus DP-71 camera. Relative densities of nerve fibers in distinct locations were semi-quantitatively evaluated in tissue sections by two independent observers. Arbitrary scoring for nerve fiber densities was defined: 0 – no fibers, 1 – single, 2 – sparse, 3 – numerous.

No healthy (control) tonsils were examined, as the aim was to compare patients with potentially different postoperative pain intensities.

To determine significant differences between the distributions of the participants’ age, pain intensities, maximum pain scores, mean day of return to a normal diet, intensity of swallowing difficulties, body weight, and number of additional doses of analgesics, the Mann–Whitney *U* rank sum test was performed with Statistica version 5 software (Statsoft, Inc. – Tulsa, OK, United States). Contingency tables were created and the chi-squared test was used to confirm differences in relative nerve fiber densities. Logistic regression was used to compare the incidence of readmission due to dehydration in both groups.

## Results

The indications for tonsillectomy allowed defining two groups of patients: (1) with a history of recurrent acute tonsillitis (34 participants), and (2) with no history of recurrent tonsillitis (27 participants: focus-tonsil – 20 and intense malodor – 7).

The participants’ age and gender distribution did not differ between the groups. The length of hospital stay did not differ either. Differences in pain intensity on postoperative days one, two, and five to 13 were non-significant (Table 1). Pain intensity on days three and four was significantly higher in the group with no history of acute tonsillitis. Differences in the following were non non-significant: pain duration, body weight loss on an empty stomach noted before and on the 10th day after the surgery, character and intensity of swallowing difficulties and their duration, as well as additional doses of analgesics. Healing of the pharyngeal mucosa also did not differ significantly between the analyzed groups.

**Table 1:** Comparison of mean values (standard deviations) of the measures observed in the groups.

Indications for tonsillectomy	Recurrent tonsillitis	Other indications	Statistics
*Pain intensity on postoperative days*			
1	2.8 (0.9)	2.7 (1.2)	NS
2	3.4 (0.9)	3.2 (1.5)	NS
3	2.7 (0.7)	4.1 (0.9)	*p* < 0.01
4	2.4 (0.8)	4.0 (0.9)	*p* < 0.01
5	2.6 (0.6)	3.5 (1.2)	NS
6	2.7 (1.1)	2.9 (1.7)	NS
7	2.0 (0.6)	2.5 (1.4)	NS
8	1.6 (0.7)	2.0 (1.1)	NS
9	1.3 (0.8)	2.1 (1.5)	NS

*Pain duration (days)*	12.4 (2.6)	11.0 (2.0)	NS
*Healing (0–4)*	1.0 (1.0)	1.2 (1.1)	NS
*Intensity of swallowing difficulties (1–4)*	1.9 (0.8)	2.2 (1.1)	NS
*Dysphagia duration (days)*	11.7 (1.8)	11.0 (2.6)	NS
*Body weight loss (kg)*	4.6 (1.4)	5.4 (3.4)	NS
*Body weight loss (%)*	7.0 (1.4)	6.5 (3.9)	NS
*Additional doses of analgesics*	5.3 (1.4)	4.6 (2.8)	NS

NS, no statistical significance; Healing, redness and edema of vast regions of pharyngeal mucosa, including the uvula, 4; edema of the uvula with or without redness and/or edema of anterior pillar mucosa, 3; redness and edema of peritonsillar tissues excluding the uvula, 2; redness of anterior pillar mucosa without edema, 1; normal mucosa, without redness or edema; Swallowing, 1 – mild swallowing disorders, drinking unchanged; 2 – moderate difficulties eating and drinking; 3 – marked difficulties eating and drinking; 4 – serious difficulties eating and drinking.

Serious postoperative complications did not occur. There was no major postoperative hemorrhage in either group that required surgical attention. Hemorrhage was considered an early one if it occurred within the first 24 postoperative hours and late when bleeding occurred after 24 h. Mild, spontaneously subsiding hemorrhage occurred in four patients, equally divided between the groups. All cases of postoperative hemorrhage occurred after 24 h from the surgery. Five patients in the group without a history of recurrent tonsillitis *vs.* one in the group with a history of recurrent tonsillitis required readmission due to dehydration (significant difference, with *p* < 0.05).

**Figure 1:**
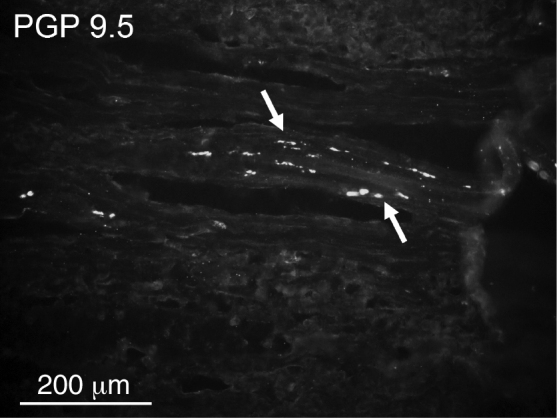
A few protein gene product (PGP) 9.5-immunoreactive nerve fibers (arrows) in the capsule of the tonsil.

**Figure 2:**
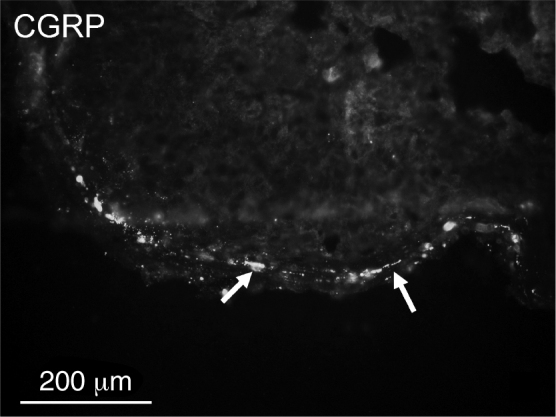
Single calcitonin gene-related peptide (CGRP)-immunoreactive nerve fibers (arrows) in the capsule of the tonsil.

**Figure 3:**
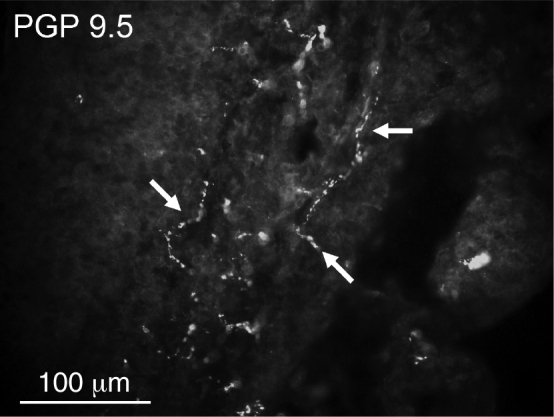
Numerous protein gene product (PGP) 9.5-immunoreactive nerve fibers (arrows) in the lymphoid tissue of the tonsil.

Overall densities of nerve fibers found in the tonsillar capsules were low. PGP 9.5-immunoreactive fibers were more numerous than CGRP-positive fibers (Table 2, Figure 1, Figure 2). CGRP fibers were found almost exclusively in the capsule (Fig. 2) while numerous PGP 9.5 fibers (mostly related to blood vessels) were very numerous in the lymphoid tissue (Fig. 3). No significant differences in densities of PGP 9.5- as well as CGRP-immunoreactive nerve fibers were observed (Table 2).

**Table 2:** Comparison of relative densities of PGP 9.5- and CGRP-immunoreactive nerve fibers.

	PGP 9.5	CGRP
Recurrent tonsillitis	2	0
Other indications	2	1
Statistics	NS	NS

PGP 9.5, protein gene product 9.5; CGRP, calcitonin gene-related peptide; NS, no statistical significance.

External validity of the findings was confirmed by the fact that all the patients gave consent to participation (there was no influence of potential differences between those who gave consent and non-consenters); the examined groups of patients were coherent in terms of age and lack of co-morbidities, and that they reflected the demographic and socioeconomic characteristics of the general population treated in a community-based hospital. Also, the surgical technique was the same in all the participants.

## Discussion

The data from this study demonstrate that post-tonsillectomy pain intensity recorded after KTP laser tonsillectomy on postoperative days three and four in patients with no history of recurrent tonsillitis was significantly higher than in those in whom recurrent tonsillitis was the indication for surgery. Also, the readmission rate due to dehydration was higher in the former group. So far, evidence concerning effects of indications for the surgery on post-tonsillectomy pain has not been reported.

Logically, postoperative pain in patients tonsillectomized due to recurrent tonsillitis should be more intense than in the other group, as dissection of the tonsils from the surrounding scarred tissues could be associated with greater intra-operative trauma. Therefore, while planning the study, it was decided to use laser instead of performing cold steel surgery, in order to eliminate potential influence of forceful dissection of the tonsils in this group of patients on the results of the study. Thus, it was possible to eliminate factors relating to the surgical procedure itself from the analysis of post-tonsillectomy outcomes. This study did not confirm more intense pain in the group of patients with recurrent tonsillitis in anamnesis, most probably because laser enables easy dissection of the tonsils from their beds.

Significant differences in reported pain intensity were observed on the days when post-tonsillectomy pain in most patients was most aggravated.^8^ In previous studies, pain scores after dissection tonsillectomy, as measured by a visual analog scale, differed significantly between every third consecutive day following postoperative day four.^9^ Two-thirds of the patients required analgesics after the first postoperative day. About 80% of the individuals returned to work within 14 days of surgery and almost all reported normal swallowing within 14 days of surgery,^9^ which is consistent with the authors’ observations relating to the laser-assisted tonsillectomy. However, this study did not confirm the observation that post-tonsillectomy pain, though initially slightly less after KTP laser than after standard cold steel tonsillectomy, became worse at two weeks after surgery in patients operated using the laser.^28^ The present results relating to wound healing and subsequent pain might be hard to compare with the results obtained in patients after tonsillectomy performed with the conventional method, due to slightly delayed wound healing after KTP laser surgery of the throat.^29^

Since no differences were established between the examined groups on histological examination, the observation that pain experienced by patients with no history of recurrent tonsillitis was significantly more intense could be explained by the fact that the patients with a history of recurrent acute tonsillitis were more familiar with this kind of pharyngeal pain, and therefore tolerated it better.

Densities and distribution of nerve fibers within the tonsillar capsule and lymphatic tissue differed between individuals and anatomical regions of the tonsil, and the distribution was dependent on the tissue intersection plane. Hence, considerable differences in densities of nerve fibers were found locally in various examined tonsils. However, the observed overall differences were statistically non-significant.

The strengths of this study include direct comparison of statistically significant groups of participants operated for the same symptoms using the same technique and with long-term follow-up. Weaknesses include the fact that there could be other factors determining differences in postoperative pain perception, not accounted for in the current study. Determining these factors would foster future research in this field. In addition, the scales used for swallowing difficulty and postoperative wound healing was not validated, and therefore subject to bias.

Several important conclusions can be drawn from this study. The results obtained will assist in preoperative counseling of patients undergoing tonsillectomy regarding possible postoperative pain intensity and establishing improved follow-up protocols. Based on the obtained results, prior to surgery detailed medical history is collected from all candidates for tonsillectomy. If a patient has no history of recurrent tonsillitis, she/he is informed about increased probability of intense postoperative pain a few days after tonsillectomy, and analgesic dosage adjustment is advocated. These patients are also advised to stay in hospital longer, until their aggravated swallowing difficulties subside, as they are at risk of re-admission due to dehydration.

## Conclusion

Patients qualified for tonsillectomy due to recurrent tonsillitis report lower pain intensity than those with other indications for the surgery. Postoperative pain intensity was unrelated to nerve fiber density.

## Conflicts of interest

The authors declare no conflicts of interest.
